# Retraction Note to: Decreased expression of the long noncoding RNA LINC00261 indicate poor prognosis in gastric cancer and suppress gastric cancer metastasis by affecting the epithelial–mesenchymal transition

**DOI:** 10.1186/s13045-017-0544-6

**Published:** 2018-01-04

**Authors:** Yu Fan, Yan-fen Wang, Hua-fang Su, Na Fang, Chen Zou, Wen-feng Li, Zheng-hua Fei

**Affiliations:** 1 Cancer Institute, The Affiliated People’s Hospital of Jiangsu University, Zhenjiang, People’s Republic of China; 2 Department of Pathology, the First People’s Hospital of Yangzhou/The Second Clinical Medical College, Yangzhou University, Yangzhou, People’s Republic of China; 3 Radiotherapy and Chemotherapy Department, The 1st Affiliated Hospital of Wenzhou Medical University, Wenzhou, China

## Retraction

The authors have retracted this article [1] because of significant overlap of text and some images with a previously published article by Xu et al. [2]. A formal investigation by the 1st Affiliated Hospital of Wenzhou Medical University has found that some panels in Fig. 6b [1] are identical with panels in Fig. 5a [2] and some images of mice lungs in Fig. 6c [1] are identical with images in Fig. 3g [2]. The data reported in this article are therefore unreliable. All authors agree to this retraction.
